# Laryngology and Rhinology

**Published:** 1900-12

**Authors:** Barclay J. Baron


### LARYNGOLOGY AND RHINOLOGY.

Several important matters were discussed at the meetings of the American Laryngological Association, May, 1900.

Dr. Blois[1] opened a discussion on the **treatment of fractures of the nose**, and advocated a bit of stiff rubber tubing, inserted by means of a well-greased pair of scissors, as an internal splint, after reduction of the dislocation. Plaster of Paris bandage makes a good external splint. Dr. Casselberry moulds the plaster of Paris over the nose, carries it back to the line of the ears, making it thinner posteriorly, and secures it by tapes passing above and below the ears behind the head. Such splints must be worn ten days or more. Internally, nosophen gauze applied under cocaine acts well. If the fracture is low down in the nose, an ordinary vulcanized tube answers well. Other speakers advocated the use of the Bernays' sponge as an internal splint, a thin metal splint or an adhesive plaster externally.

Dr. Emil Mayer described a case of **recurring membranous faucitis**, due to the bacillus of Friedlander. This bacillus has been found in local manifestations in stomatitis, ozœna, rhinoscleroma, acute suppurative rhinitis; in pus in the antrum of Highmore; in membranous bronchitis and other lung diseases; in pericarditis, pyelo-nephritis, meningitis, parotiditis, otitis, and generally in pyæmia and septicæmia.

The patient had had for eighteen months a membrane formed either over the entire pharynx and soft palate, or over the latter alone, and had never been free from it for more than fourteen consecutive days during this time. It formed and exfoliated on the third day, when the throat appeared normal. If it was prematurely removed, a raw surface, bleeding on manipulation, was present. The pieces that exfoliated were pearly white, with pinhole perforations. Constitutional manifestations were very slight. Examination of the membrane showed it to consist of squamous epithelium arranged in layers three to six cells in thickness. On the surface of the membrane, between the cells as well as within them, are very numerous short bacilli. There is no fibrin present, nor can leucocytes, blood-vessels, or connective tissue be detected.

Dr. J. N. Mackenzie opened a discussion on the early **diagnosis and treatment of laryngeal cancer.** There are three methods of arriving at a diagnosis:—

1. The naked-eye appearances, combined with the clinical history.
2. Thyrotomy.
3. Microscopic examination of a removed fragment.

[1] *J. Laryngol.*, 1900, xv. 298.

The first and second frequently go together, and a more extensive division of the cervical tissues than a mere laryngofissure is allowable in cases of reasonable doubt, or where one cannot otherwise define the exact territory occupied by the disease.

As regards the removal of a piece for microscopic examination, it is a dangerous procedure, as it renders the patient liable to auto-infection or of metastasis. If reasonable doubt exists, it should not be done. Early and thorough removal is necessary, and as regards operation on one half of the larynx, the other half that is left is not of much value for voice production. Intra-laryngeal operations in extensive disease are to be condemned. Simple thyrotomy with curettage is a reversion to the status of fifty years ago.

Delavan remarked that of 163 cases occurring in the practice of eight Continental surgeons who had had at least ten personal cases on which radical operations were performed, only 6 per cent. of recoveries took place.

Solis-Cohen called attention to the following points in the performance of laryngectomy:—

1. Operate with the head in a semi-inverted position, to prevent entry of septic matter into the lungs.

2. Preliminary tracheotomy should be done, otherwise we are troubled by the descent of the trachea.

3. If possible, retain the epiglottis.

4. Shut off all communication of the mouth with the air passages. In attaching the upper part of the trachea to the skin, the tube should be slit longitudinally for a short distance.

5. All dressings should be avoided. Feed by enema, and use no tube by the mouth. Allow no packing, as it causes constant desire to swallow.

6. Remove the larynx from below upward, and elevate the foot of the bed after operation.

**Secondary hemorrhage after the use of suprarenal extract.**

Hopkins finds that suprarenal extract is apt to cause hemorrhage, and considers that we should always use intranasal packing after its use in operations on the nose.

Swain had seen more hemorrhage when the extract and cocaine are used in combination than when either of them is used alone.

Farlow had not seen hemorrhage occur after its use, but had had some remarkable cures of coryza from it.

Grünbaum[1] calls attention to the fact that suprarenal gland extract does not cause coagulation, and so does not seal vessels with clots. From this it follows that unless the application is repeated we may get fresh bleeding. Hemorrhage from a small artery is more likely to be affected by it than capillary oozing from

[1] *Brit. M. J.*, 1900, ii. 1307.

a hyperæmic mucous membrane, since it acts by causing contraction of the plain muscle fibres of blood-vessels.

Lermoyez has written a paper on the **treatment of nasal hydrorrhœa** with atropine and strychnine.[1]

He believes that the fluid that issues from the nose is a true secretion and not a mere exosmosis, and is due to abnormal excitation of the secretory filaments of the superior maxillary nerve. The obstructive phenomena are considered to be due to true vaso-dilator activity rather than to paralysis of vaso-constrictors. The local lesions in the nose may be, and often are, secondary and not causal. The hyperæsthetic spots are situated at points especially liable to be irritated by inspired particles and by the pressure resulting from hyperæmic swelling.

Leaving aside all local treatment by cautery, snare or knife, we are recommended to attack the hyper-secretion with atropine and the vaso-dilatation with strychnine. Moderate doses taken once a day for ten days and twice daily for the next ten days, with a fortnight's rest after the twentieth day, usually suffice to really cure this troublesome condition.

Only occasionally is it necessary to administer the drugs three times a day for ten days after the twentieth day.

BARCLAY J. BARON.

